# The Role of Modern Diagnostic Imaging in Diagnosing and Differentiating Kidney Diseases in Children

**DOI:** 10.34763/devperiodmed.20182201.8187

**Published:** 2018-04-12

**Authors:** Artur Maliborski, Arkadiusz Zegadło, Małgorzata Placzyńska, Małgorzata Sopińska, Marianna Lichosik, Katarzyna Jobs

**Affiliations:** 1 Department of Medical Radiology, Military Institute of Medicine, Warsaw, Poland; 2 Department of Paediatrics, Nephrology and Allergology, Military Institute of Medicine, Warsaw, Poland

**Keywords:** diagnostic imaging, children, urinary tract diseases, diagnostyka obrazowa, dzieci, choroby układu moczowego

## Abstract

Urinary tract diseases are in the group of the most commonly diagnosed medical conditions in pediatric patients. Many diseases with different etiologies are accompanied by pain, fever, hematuria, or urinary tract dysfunction. Those most common ones in children are urinary tract infections and congenital malformation. They can also represent tumors or changes caused by systemic diseases. Clinical tests and even more often additional imaging studies are required to make a proper diagnosis of urinary tract diseases. Just a few decades ago urography, cystography or voiding cystourethrography were the main methods in diagnostic imaging of the urinary tract. Today’s imaging methods supported by digital radiographic and fluoroscopy systems, high sensitivity detectors with quantum detection, advanced algorithms eliminating motion artifacts, modern medical imaging monitors with a resolution of three or even eight megapixels significantly differ from conventional radiographic methods. The methods that are currently usually performed are: computed tomography, magnetic resonance imaging, isotopic methods and ultrasonography using elastography and new solutions in Doppler imaging.

Modern techniques are currently focused on reducing radiation exposure with better imaging capabilities. The development of these techniques became an essential diagnostic aid in nephrological and urological practice. The aim of this paper is to present the latest solutions that are currently used in the diagnostic imaging of urinary tract diseases.

## Streszczenie

Choroby układu moczowego należą do grupy najczęściej diagnozowanych w populacji dziecięcej. Dolegliwości bólowe, gorączka, krwiomocz lub dysfunkcja układu moczowego towarzyszą wielu chorobom o różnej etiologii. U dzieci najczęściej są wynikiem zakażeń układu moczowego oraz wad wrodzonych. Mogą być również wynikiem nowotworów lub zmian zachodzących w wyniku chorób układowych. W prawidłowo prowadzonym procesie diagnostycznym chorób układu moczowego niezbędne jest wykonanie testów klinicznych oraz coraz częściej dodatkowych badań obrazowych. Badanie metodą urografii, cystografii lub cystouretrografii mikcyjnej, pozostawało jeszcze kilkanaście lat temu główną metodą w diagnostyce obrazowej układu moczowego. Dziś metody te, wspomagane przez systemy radiografii i fluoroskopii cyfrowej, bardzo czułe detektory z kwantową wydajnością detekcji, zaawansowane algorytmy redukcji szumów, nowoczesne środki cieniujące i monitory do analizy obrazów medycznych z rozdzielczością trzech a nawet ośmiu megapikseli znacząco różnią się od możliwości ówczesnej konwencjonalnej radiografii. Obecnie często stosuje się obrazowanie przy wykorzystaniu takich technik jak: tomografia komputerowa, rezonans magnetyczny, metody izotopowe, a także ultrasonografia z wykorzystaniem elastografii i nowych rozwiązań w obrazowaniu dopplerowskim.

Nowoczesne techniki badań są aktualnie nastawione na ograniczanie promieniowania rentgenowskiego przy coraz lepszych możliwościach obrazowania. Rozwój tych technik stał się niezbędną pomocą diagnostyczną w praktyce nefro- i urologicznej. Celem niniejszego opracowania jest prezentacja najnowszych rozwiązań stosowanych obecnie w diagnostyce obrazowej chorób układu moczowego.

Urinary tract diseases are in the group of the most commonly diagnosed medical conditions both in adults and in pediatric patients. The most common ones in this group are urinary tract infections, which affect over 150 million people each year. Urinary tract infections in children account for about 1% of all pediatric office encounters and 5 to 14% of pediatric emergency department visits [1, 2]. In small children (1-3 years of age) with febrile urinary tract infection, the inflammatory process in most cases more or less involves renal parenchyma [3]. Vesicoureteral reflux is present in about 30% of the children presenting with febrile urinary tract infection. This has been associated with increased risk of infections and can result from abnormal urinary tract anatomy [4]. Blockage in urinary flow, whether caused by an organic cause, malformations or a functional cause, might be an additional risk factor for infection.

Another group of urinary tract disorders in children are changes in the structure of kidney parenchyma, including post-inflammatory changes resulting from systemic diseases and focal lesions which might be mild or malignant, acquired or congenital. These processes can lead to renal failure or complications, such as dysfunctions of other organs [5, 6, 7].

The correct therapeutic process depends on identifying the causes of the urinary tract diseases and differential diagnosis of the abnormalities detected. In cases when clinical diagnosis is doubtful or there are recurrent infections, hematuria, fever of unknown origin, pain (renal colic), additional tests including radiology and imaging diagnostics are required.

Among many available imaging methods in urinary tract evaluation, radiography, ultrasonography, computed tomography, the most commonly used ones are magnetic resonance imaging and nuclear medicine methods. Dynamic technological progress in biomedical engineering is noticeable in all the disciplines of medicine but it is particularly evident in radiology and imaging diagnostics. Advanced algorithms of digital restoration and the high quality of modern contrast agents enable an accurate diagnosis of very fine structures, such as the urinary tract or small renal vessels. They also make it possible to perform functional tests, such as renal perfusion, with fewer side effects. In order to correctly perform specialized tests and to properly interpret the images obtained by the physician, it is necessary to have a knowledge about the condition itself, about how to select appropriate examinations, and to obtain proficiency and experience in the diagnostic procedure, as well as closely cooperate with clinicians.

Cystography and voiding cystourethrography performed by means of standard radiological procedure using iodinated contrast agents remain methods of choice in lower urinary tract diagnostics. Modern fluoroscopy X-ray imaging systems allow precise imaging of the urinary tract using very low radiation doses. Currently the most commonly used method of visualization of the urinary tract in children is pulsed fluoroscopy (3-7 pulses per second) with a lower radiation dose than radiographic exposure, in accordance with the ALARA principle (as low as reasonably achievable). Reducing the field size of radiation exposure to the area of interest (blending) and shortening the time of exposure should be applied. Performing cystourethrography or cystography by modern imaging devices according to the protocols adjusted to the size of a child makes it possible to achieve a very low effective dose of 0.024mSv. A study comparing radiation exposure between standard cystourethrography and direct isotope cystography showed a significantly lower radiation burden of fluoroscopic cystography with 0.024 mSv vs. 0.23 mSv [8]. The effective dose in both cases remained well below the annual background radiation dose of 2.4 mSv. At the same time the total exposure time was 7s, whereas in direct isotope cystography the isotope effect on the bladder wall was on average 38 min. In addition, fluoroscopy shows more anatomical details, especially in the case of posterior urethral valves [8]. Modern pulsed fluoroscopy systems also enable loop recording (film recording) that can be replayed and then reanalyzed to assess urinary tract function (e.g. ureteral peristaltic wave) retrospectively after the study is completed. The purpose of voiding cystourethrography is to assess the bladder wall, bladder capacity and emptying capability, the presence of vesicoureteral reflux and urethra defects including posterior urethral valves.

Standard intravenous urography enables us to visualize the pyelocalyceal system, ureteral peristalsis and to evaluate the time and dynamics of parenchymal excretory function. However, this method is very limited in showing renal cortical defects [9, 10].

The first work on ultrasound in medicine was initiated in the 1950s. Today, after more than sixty years of technological progress, this method is one of the basic tools of imaging diagnostics. Ultrasonography based on the emission and detection of reflected sound waves, free of harmful effects of ionizing radiation, is a particularly desirable method in pediatric diagnostics. It is characterized by moderate contrast resolution and the highest spatial resolution of all the imaging methods and makes it possible to visualize very small structures [9]. It is a method of choice to diagnose hydronephrosis and is also highly useful as a screening method for renal cancer, but it is limited in detecting uroepithelial tumors [10, 11]. One of the latest ultrasound applications in the diagnostics of the urinary tract in children is voiding urosonography. A study comparing standard fluoroscopic voiding cystourethrography to voiding urosonography showed considerable agreement in the diagnosis, confirmed by intraoperative surgical observation in children aged 5 months to 13 years. In urosonography second-generation ultrasound contrast agents are used to assess the bladder, vesicoureteral reflux with accurate imaging of renal calyces in high-grade reflux and to diagnose posterior urethral valves. The use of 5-6 MHz convex probes with harmonic imaging makes it possible to visualize structures in presentation B with contrast agent enhancement of the urinary tract. A linear probe can be used to achieve a higher spatial resolution [12]. It is advised to use contrast agent imaging settings which extend the enhancement properties of the agent. Some researchers indicate the higher sensitivity of urosonography in detecting low grade refluxes than standard fluoroscopic cystourethrography because of constant, non-pulse and non-intermittent observation. The ultrasound probe acquires echoes even from a very small number of contrast microspheres that pass into the ureter. The high cost of contrast agents can be compensated by their low consumption - one vial provides several tests after dilution of the agent in a physiological saline solution. Previously, since 2001, the registration of these agents allowed them to be used in the urinary tract in adults. A special Uroradiology Task Force of the European Society of Pediatric Radiology confirmed in 2011 the safety of more than 4,000 intravesical applications of ultrasound contrast agents in children [13, 14, 15, 16].

Over the past ten years the dynamic development of ultrasound hardware, progress in constructing ultrasonographic probes and computer applications have enabled the implementation of new ultrasound imaging techniques. This is primarily elastography, which had its initial uses in imaging liver and superficial organs. There are currently attempts to apply this method in nephrological diagnostics. Another area of development is the significant increase of the sensitivity of Doppler techniques in recent years, allowing the visualization of low-velocity flows, also in vessels small in diameter.

The improvement of ultrasonographic computational capacity and the progress in the construction of probes made it possible to simultaneously process more data and forms of ultrasound beams with different shapes and frequencies. Previously this was impossible. Such development resulted in the introduction of new solutions in Doppler imaging which visualizes blood flow. One of the latest solutions available on the market is Ultrafast Doppler. This technique allows simultaneous color and spectral flow analysis and quantification. Moreover, in contrast to the solutions used so far, it allows to acquire image information at several frames at the same time. It offers the ability to compare the differences in the time and speed of blood flow from various arterial segments, e.g. the difference between the renal artery and interlobar arteries.

**Fig. 1:**
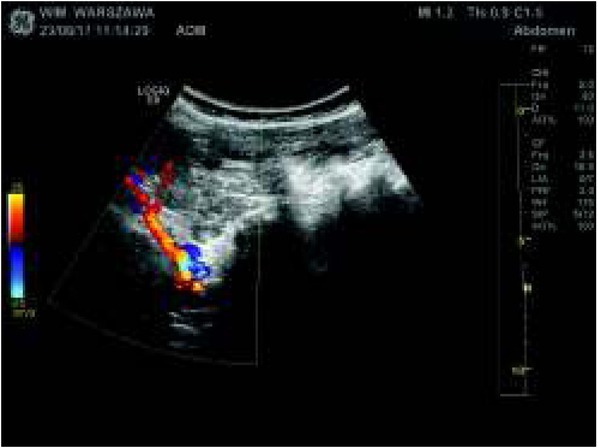
Doppler imaging of renal artery. Ryc. 1. Obrazowanie metodą kolorowego dopplera głównego pnia tętnicy nerkowej.

Ultrafast Doppler imaging introduced by different companies allows us to visualize lower blood flow velocity in vessels of smaller diameter. This is especially important for imaging the blood flow in the renal parenchyma that changes due to glomerulopathy or other systemic diseases involving renal parenchyma, including the renal cortex. Superb Microvascular Imaging (SMI) is a new Doppler technology that improves the detection of microvascular flow in superficial organs (linear probes) and deeper structures (convex probes). This technology is based on special software solutions that can reveal low signal levels with high refresh rates while eliminating motion artifacts [17]. New processing algorithms and the ability to detect low-velocity blood flows at the suppression of movement signals also provide the basis to refining previous Doppler imaging techniques which allow users to benefit from a greater visualization of renal arteries with suspected stenosis. Good imaging and a high refresh rate make it possible not only to evaluate the morphology but also the parameters of blood flow. (Figure 1).

Computed tomography and magnetic resonance imaging are an important complement to the diagnostic imaging of kidney diseases. Computed tomography with special dose reduced protocols plays an increasingly important role in detecting calcified stones. The effective radiation dose for computed tomography of the urinary tract to detect stones using the standard protocol was between 4.3 and 16.1 mS. By applying special protocols using dose reduction algorithms the effective radiation dose varied between 0.5 mSv and 2.82 mSv. Dose reduction algorithms make it possible to use a lower load of radiation than the annual exposure to natural background radiation [18, 19].

**Fig. 2:**
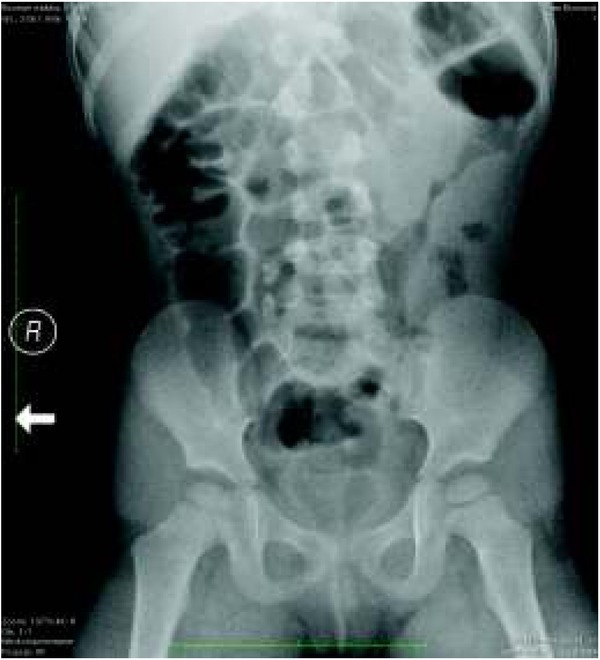
Abdominal X-ray. Two stones in the right ureter and suspected right renal pelvic stone. Ryc. 2. Zdjęcie przeglądowe jamy brzusznej. Widoczne dwa złogi w moczowodzie prawym i podejrzenie złogu w miedniczce nerki prawej.

**Fig. 3:**
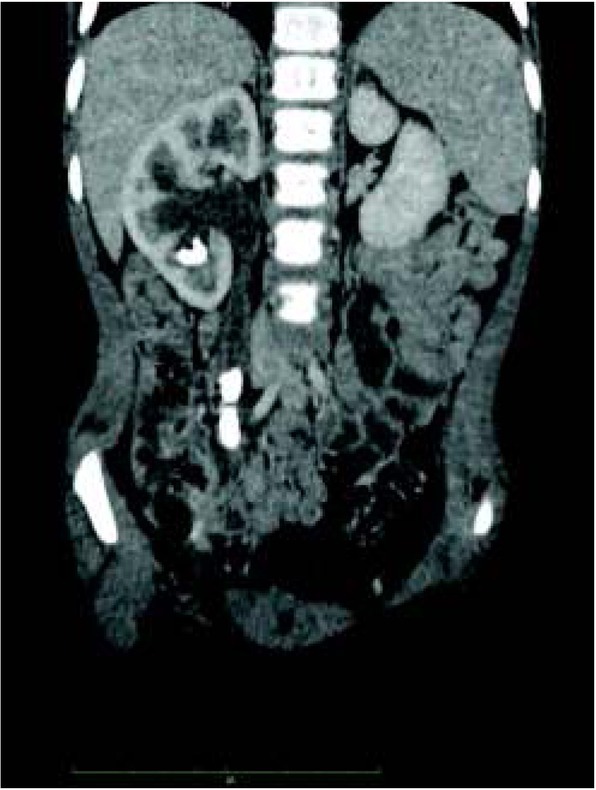
Contrast enhanced CT, corticomedullary phase. Two stones in right ureter and right renal pelvis. Ryc. 3. Tomografia komputerowa ze wzmocnieniem kontrastowym, faza miąższowa. Widoczne złogi w prawym moczowodzie i miedniczce nerki prawej.

**Fig. 4:**
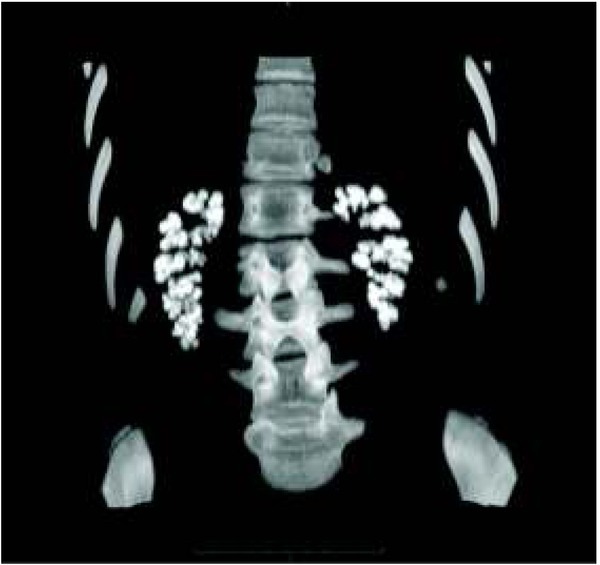
Nephrocalcinosis. Ryc. 4. Nefrokalcynoza.

The introduction of spectral scanners with atomic number measurements enables precise urinary stone characterization and the determination of their chemical composition. This helps to select the optimal treatment method. Computed tomography also allows the differentiation of nephrocalcinosis from pelvic and ureteral stones (Figure 2, Figure 3, Figure 4) [20, 21].

**Fig. 5:**
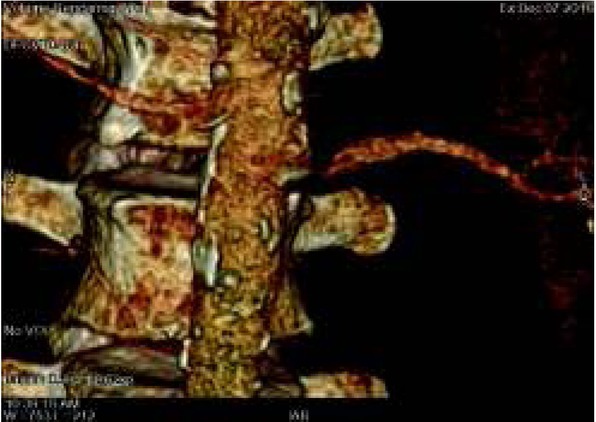
CT Angiography. Left renal artery stenosis. Ryc. 5. Angio CT. Zwężenie lewej tętnicy nerkowej w miejscu odejścia od aorty.

**Fig. 6:**
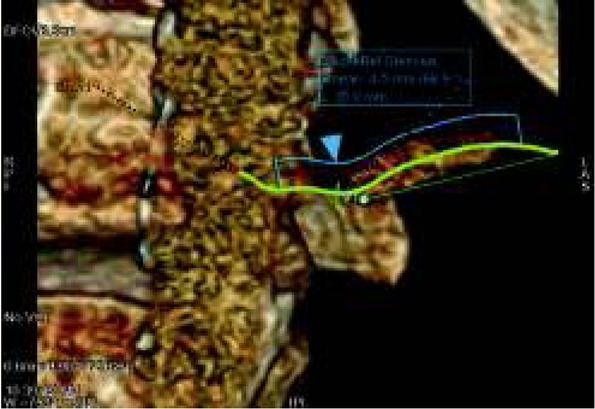
CT Angiography. Left renal artery stenosis. Measurement of arterial stenosis. Ryc. 6. Angio CT. Zwężenie lewej tętnicy nerkowej w miejscu odejścia od aorty, pomiar zwężenia światła naczynia.

Angio-CT examination remains the gold standard in vascular imaging. The unclear picture of renal arteries or suspicion of renal artery stenosis in ultrasonography is an indication for angio-CT testing which allows scientists to make accurate measurements of the degree of arterial stenosis (Figure 5, Figure 6).

In doubtful cases, a CT urography, where the ureters are contrasted enables users to visualize its sinous course or filling defects caused by ureteral stones.

**Fig. 7:**
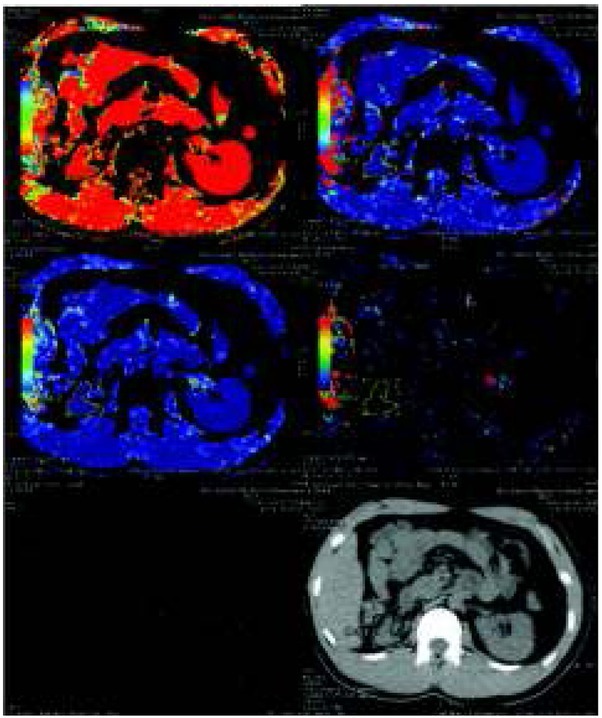
Renal perfusion study with reduced perfusion of right kidney. Ryc. 7. Badanie perfuzyjne nerek obrazuje obniżoną perfuzję nerki prawej.

CT scans with contrast enhancement makes it possible to evaluate renal parenchyma perfusion with a small amount of contrast agent (40 ml). Renal parenchymal perfusion is affected by inflammation, fibrosis, renal infarction and all cases of hemodynamically relevant stenosis of renal vessels. The chronic process can lead to ischemic nephropathy, i.e. kidney damage resulting from its reduced perfusion (Figure 7).

Evaluation of organ perfusion is based on the analysis of the amount of blood entering, blood leaving, flow time and blood volume in the imaged area. The obtained values can be represented by color coding or in numerical form.

**Fig. 8:**
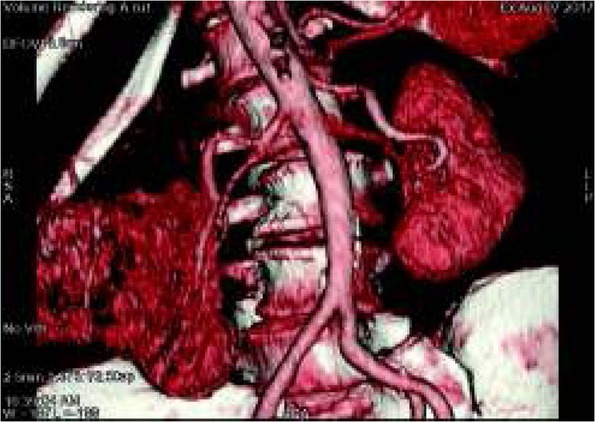
Abdominal enhanced CT. Volumetric reconstruction. Right kidney cancer. Ryc. 8. Badanie CT jamy brzusznej po wzmocnieniu kontrastem, rekonstrukcja objętościowa. Rak nerki prawej.

Highly vascularized proliferative lesions affecting kidney parenchyma are strongly enhanced after contrast administration. Lesions with a small number of vessels are poorly contrasted against enhanced renal parenchyma. Volumetric reconstruction allows the assessment of the spatial location of the proliferative lesion (Figure 8).

Magnetic resonance imaging (MRI) of the urinary tract in children is a valuable supplement and an alternative to X-ray methods and ultrasonography. The significantly higher safety profile of this method and lack of ionizing radiation are the reasons this is a preferable method in children. The limitations of its application are: a relatively long scan time: depending on the test type from 30 to 60 minutes, and the need to remain stationary during the test. Therefore, the use of magnetic resonance imaging in children under 12 years of age usually requires general anesthesia. Because of the highest contrast resolution of all the imaging methods and the ability to repeat the study in multiple sequences without fear of radiation exposure, MRI studies are particularly useful for differentiating focal lesions located in the renal parenchyma, as well as in the pyelocalyceal system [10, 11]. These tests are usually performed using a paramagnetic contrast agent, except in situations where it is contraindicated (contrast allergy, renal failure). In case of the dilatation of the urinary tract, the use of T2-weighted images clearly shows their structure. In other cases, it is very useful to use T1-weighted sequences after administration of the contrast agent in the delayed phase, called the urographic phase. Inflammatory and posttraumatic changes, including edema, may be well visualized in MRI studies. Dynamic studies with organ perfusion assessment may also be performed in magnetic resonance, similarly to computed tomography, but with no exposure to ionizing radiation. Currently magnetic resonance imaging is most commonly used to differentiate renal focal lesions that cannot be unquestionably evaluated by other imaging methods.

**Fig. 9:**
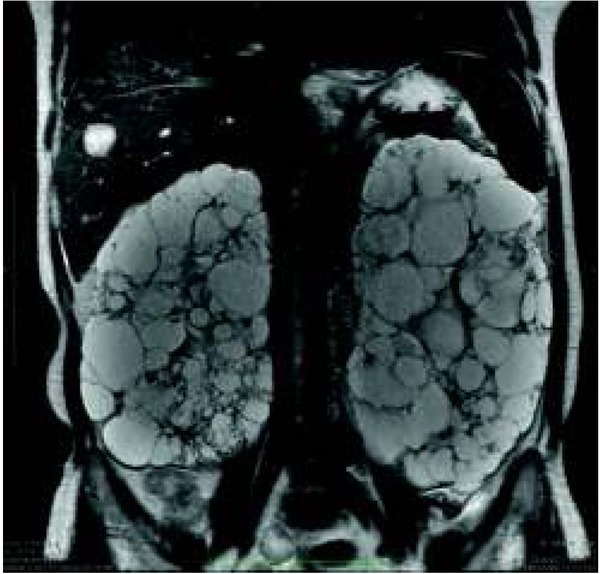
Magnetic resonance imaging, T2-weighted images. Polycystic kidney disease. Cyst in the liver. Ryc. 9. Obrazowanie metodą rezonansu magnetycznego, obrazy T2 zależne. Wielotorbielowatość nerek. Zmiana torbielowata wątroby.

It relatively often concerns cystic lesions in the kidney with thick enhanced walls that may cause diagnostic difficulties [22] (Figure 9).

The last group of imaging methods used in the diagnosis of urinary tract diseases are isotope studies (renoscintygraphy). They play an important role in the diagnostics in children, are less invasive, and the degree of radiation is often lower than in radiological tests. Scintigraphy is an imaging method used to evaluate the structure and function of kidneys, in which a substance containing a radioactive isotope (radiotracer) is applied. The isotope emits radiation that can be measured and is thus used to evaluate organs of the body and their function. The most commonly used isotopes are technet 99 and iodine 131. By selecting suitable methods and radioactive markers, renal perfusion, glomerular filtration rate, tubular secretion and urine output can be assesed. In some cases the renal isotope scan is also supplemented by pharmacological tests to evaluate renal function after captopril or furosemide administration.

The renoscintigraphic study is performed as a static and dynamic-functional test. Static renal scintigraphy is a study that shows the location, mobility, size and shape of the kidney. Based on the distribution of the isotope, various changes in the kidney can be detected, such as scars, cysts, abscesses, tumors or anomalies. This test uses the 99mTc-DMSA marker which is excreted by the kidney primarily by tubular secretion. The main advantage of this radiopharmaceutical which enables good visualization of functioning renal parenchyma is its long accumulation in the renal tubular epithelial cells. After the radioactive marker is excreted by filtration, the functioning areas of the renal parenchyma are visualized thanks to the tracer retention in the renal tubular epithelium. Photopenic areas due to reduced radiotracer uptake represent renal inflammation or scarring.

Dynamic scintigraphy (renoscintygraphy) is a study that records the movement of a marker from the time of intravenous administration to the moment of excretion into the bladder. It is used to assess the size and location of the kidneys, renal parenchyma damage (inflammatory and post-inflammatory lesions), upper urinary tract urine flow disorders and bladder emptying. It can also show perfusion disorders. Indications for performing a dynamic test are various conditions requiring functional assessment. One of them is suspected renal artery stenosis as a cause of renovascular hypertension. Dynamic scintigraphy is also used to evaluate and control renal function after acute diseases and surgical procedures (also in case of a transplanted kidney). Other indications may be suspected hypoplasia, aplasia or atrophy of one of the kidneys, as well as suspected urinary flow blockage caused by urinary stones, renal papillary necrosis or postinflammatory urinary tract obstruction. According to some authors, the study may also be useful in the evaluation of vesicoureteral reflux. The most commonly used radiopharmaceutical is 99m-Tc-DTPA (technetium-99m-labeled diethylenetriaminepentaacetate). Another isotope used is 99m-Tc-MAG3 (technetium-99m-labeled mercaptoacetyltriglycine). Ethylenedicysteine (EC) labeled with technetium-99m (99mTc) is becoming more and more popular recently. This compound is physiologically taken up by the cells of the kidney parenchyma, then it is being secreted into the urine and finally excreted in the urine through the urinary tract. Renal radiotracer activity registered with a gamma camera as scintigraphic images enables non-invasive evaluation of the function of each kidney. The percentage uptake for each kidney corresponds to a relative contribution to the renal clearance, where 100% is the clearance capacity of both kidneys. Renal relative function of 40-55% was accepted as normal, below 40% - reduced, above 55% as elevated. Renogram curves registered from each kidney for 20 minutes are the basis of dynamic scintigraphy evaluation. There are 3 phases of the renogram: vascular − renal blood flow, parenchymal (secretory) − transit time and excretory − urinary flow outside the kidney.

In order to increase the sensitivity and specificity of the test, it can be performed with a single-dose of a diuretic (Furosemide). The aim of a diuretic renal scan is to increase urine flow and therefore accelerate the removal of the radiotracer from the kidney. This study enables us to perform a more accurate evaluation of urinary flow disorders and differentiates total from partial obstruction. This test is used to assess hydronephrosis and ureteropelvic junction obstruction. No response to Furosemide or only partial response confirms the diagnosis of urinary tract obstruction with completely or partially blocked urine flow.

For the diagnosis of renovascular hypertension the captopril renal scan is performed. It consists of dynamic renal scintigraphy 20 min after oral administration of captopril. This makes it possible to better visualize the differences in blood supply and secretion between the kidneys, which is indicative of renovascular hypertension [23, 24, 25, 26].

It is estimated that renal scintigraphy (Tc-99m) is associated with an average absorbed radiation dose of 0.7 mSv (according to the “Guidelines for physicians referring for radiology services”, National Radiological Protection Center).

## Conclusions

The range of currently performed imaging methods demonstrated above is an essential diagnostic aid in nephrological and urological practice. Modern techniques are currently focused on reducing radiation exposure with better imaging capabilities.
